# Supplementary material for: CD8+ regulatory T cells are critical in prevention of autoimmune-mediated diabetes
Source: Nat Commun. 2020 Apr 22;11:1922. doi: 10.1038/s41467-020-15857-x (PMC7176710; doi:10.1038/s41467-020-15857-x)
Supplement: Supplementary file 1 — Supplementary Information [file 41467_2020_15857_MOESM1_ESM.pdf]

CD8$^+$ regulatory T cells play a critical role in prevention of autoimmune-mediated diabetes

Shimokawa et al.

Supplementary Figure 1

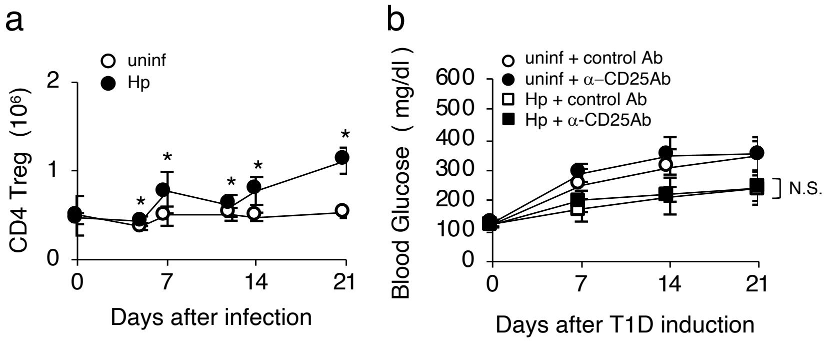

Supplementary Figure 1. No significant role of CD4Tregs in suppression of T1D in mice infected with Hp. (**a**) Absolute numbers of splenic CD4Tregs defined as $CD4^+CD25^+Foxp3^+$ cells were calculated based on flow cytometric analyses. (**b**) Hp-infected mice depleted of CD4Tregs by administration of an anti-CD25 antibody were used for induction of T1D. Blood glucose concentrations were monitored as described in **Fig. 1a**. Values represent the mean $\pm$ S.D. of 10 mice. Asterisks denote statistical significance at $p < 0.05$ calculated by two-way ANOVA . NS indicates non-significant. All experiments were repeated at least three times with similar results.

Supplementary Figure 2

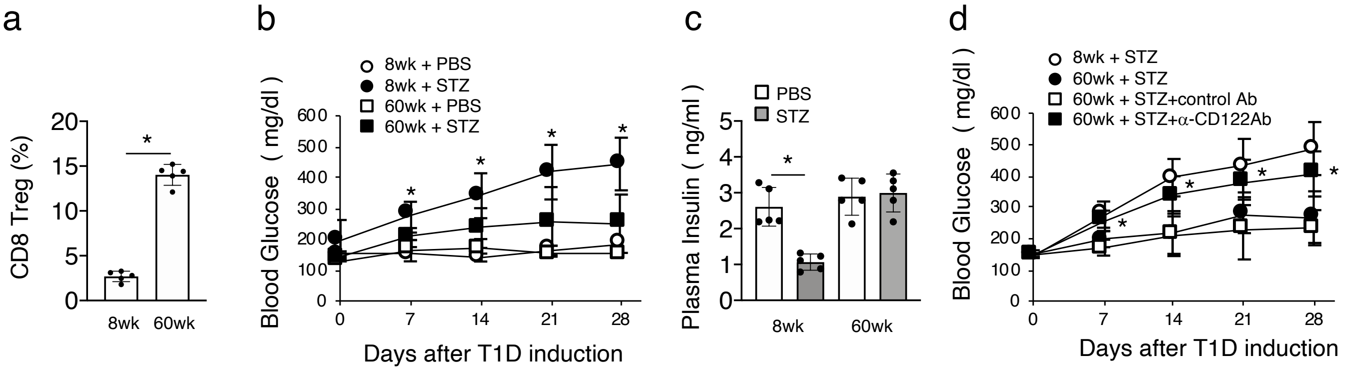

Supplementary Figure 2. Resistance to induction of T1D of aged mice. (**a**) Splenic CD8Tregs in 8- and 60-week-old mice were analysed as described in **Fig. 1d**, and the frequency is shown. Mice were treated with STZ, and blood glucose concentrations (**b**) and plasma insulin (**c**) were measured as described in **Fig. 1a, b**. (**d**) Sixty-week-old mice depleted of CD8Tregs were used for T1D induction, and blood glucose concentrations were monitored. Values represent the mean ± S.D. of 10 mice. Asterisks denote statistical significance at $p < 0.05$ calculated by the two-sided unpaired Student's *t*-test(**a**), the two-way ANOVA (**b**, **d**) Tukey post-hoc analysis (**c**). NS indicates non-significant. All experiments were repeated four times with similar results.

Supplementary Figure 3

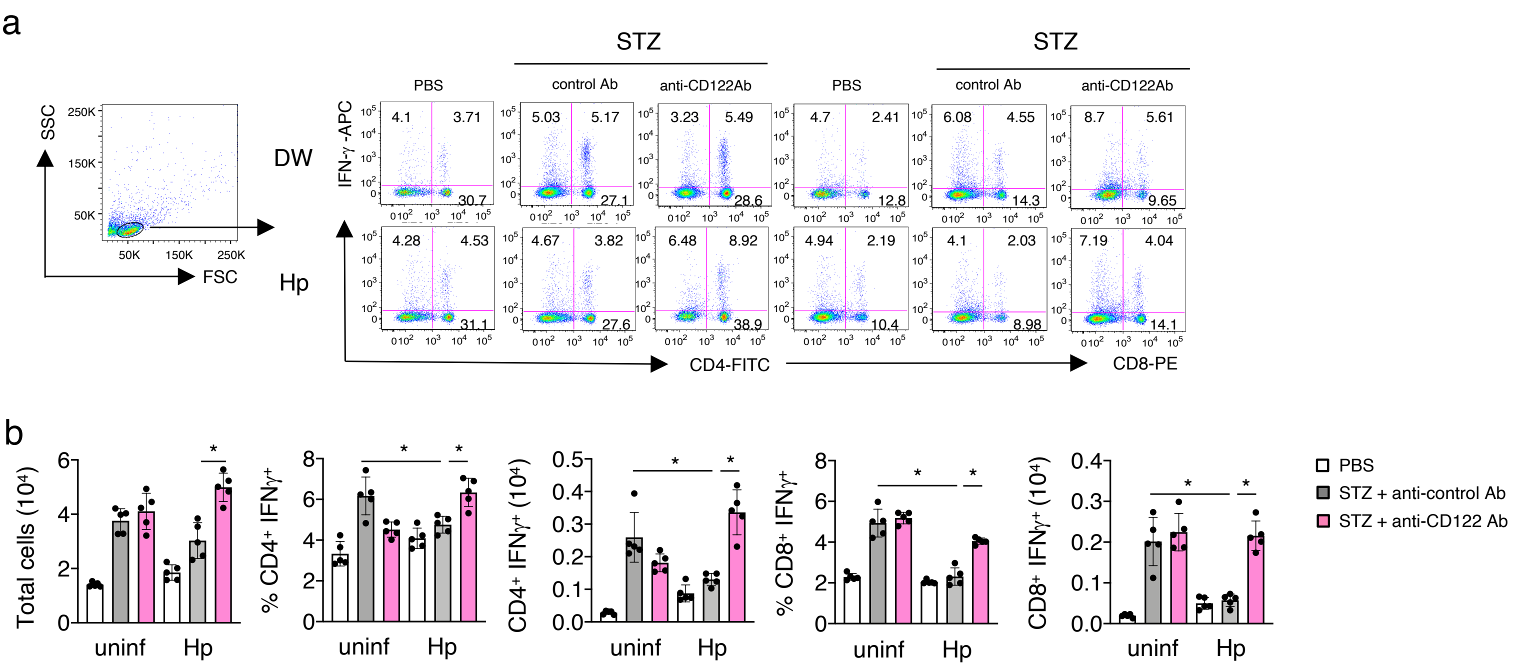

Supplementary Figure 3. *In situ* uppression of IFN-γ-producing T cells in CD8+ Treg cells-dependent manner during Hp infection. (a) Pancreatic mononuclear cells isolated from the indicated mice were analysed for production of IFN-γ after stimulation with a plate-bound anti-CD3 antibody by flow cytometry**.** (**b**)Numbers of total recovered cells, and the frequency and number of IFN-γ-producing CD4+ and CD8+ T cells are calculated. Values represent the mean ± SD from 5 mice. Asterisks denote statistical significance at $p < 0.05$ calculated by the Tukey post-hoc analysis. All experiments were repeated at least three times with similar results.

Supplementary Figure 4

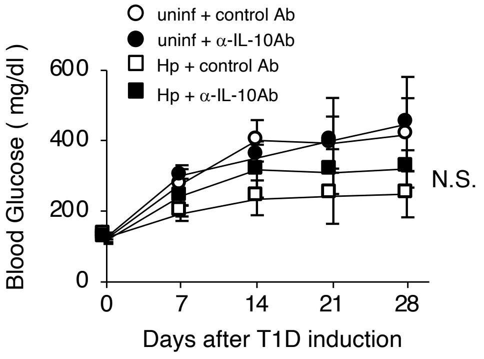

Supplementary Figure 4. Partial contribution of IL-10 to prevention of T1D in Hp-infected mice. Blood glucose levels were monitored in Hp-infected mice administered an anti-IL-10 antibody. Values represent the mean ± SD of 10 mice. NS indicates non-significant using two-way ANOVA . All experiments were repeated two times with similar results.

Supplementary Figure 5

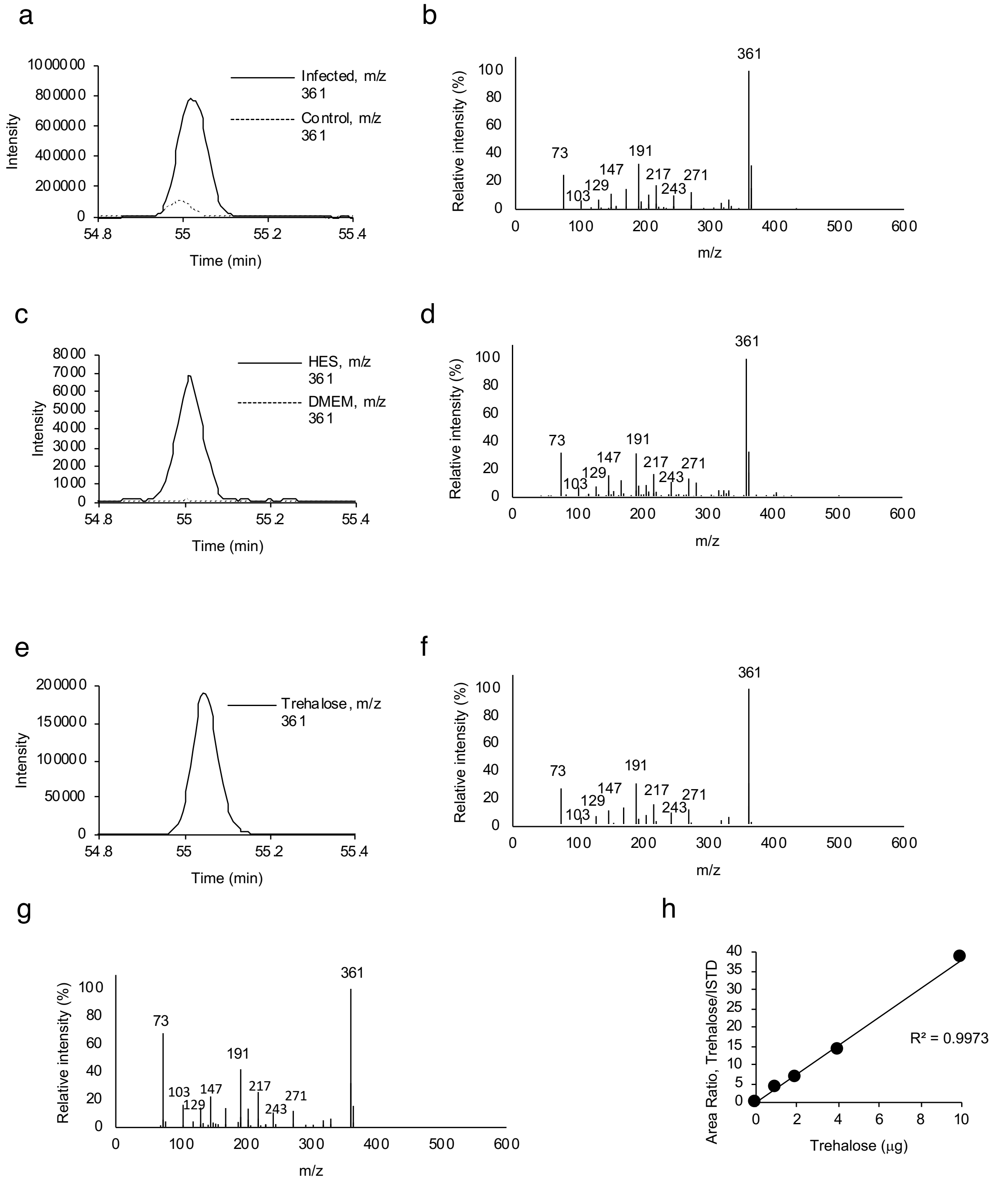

Supplementary Figure 5. Identification of trehalose in the intestinal contents and among HES antigens. Intestinal contents of infected and control mice (**a**), HES antigens, DMEM (**c**), and 50 ng trehalose (**e**) were analysed using the GC/MS full-scan mode (m/z 45 - 600) without methoximation. Representative mass chromatograms (m/z 361) showed that a compound equivalent to the peak was increased in mice infected with Hp and in HES antigens. A mass chromatogram (m/z 361) showed that the retention times were very similar among **a**, **c**, and **e**, and the mass spectrum of the peak corresponding to the infected sample in **a** (**b**) and to HES antigens in **c** (**d**) had high similarity to that of trehalose (**f**), suggesting that these peaks were derived from trehalose. (**g**) Standard mass spectrum of trehalose-8TMS collected in the spectrum library of the GCMS solution based on NIST11. (**h**) Trehalose was analysed using the GC/MS MRM mode (m/z 361 > 73) without methoximation. 2-IPM (0.5 μg) was used for the internal standard. All experiments were repeated two times with similar results.

Supplementary Figure 6

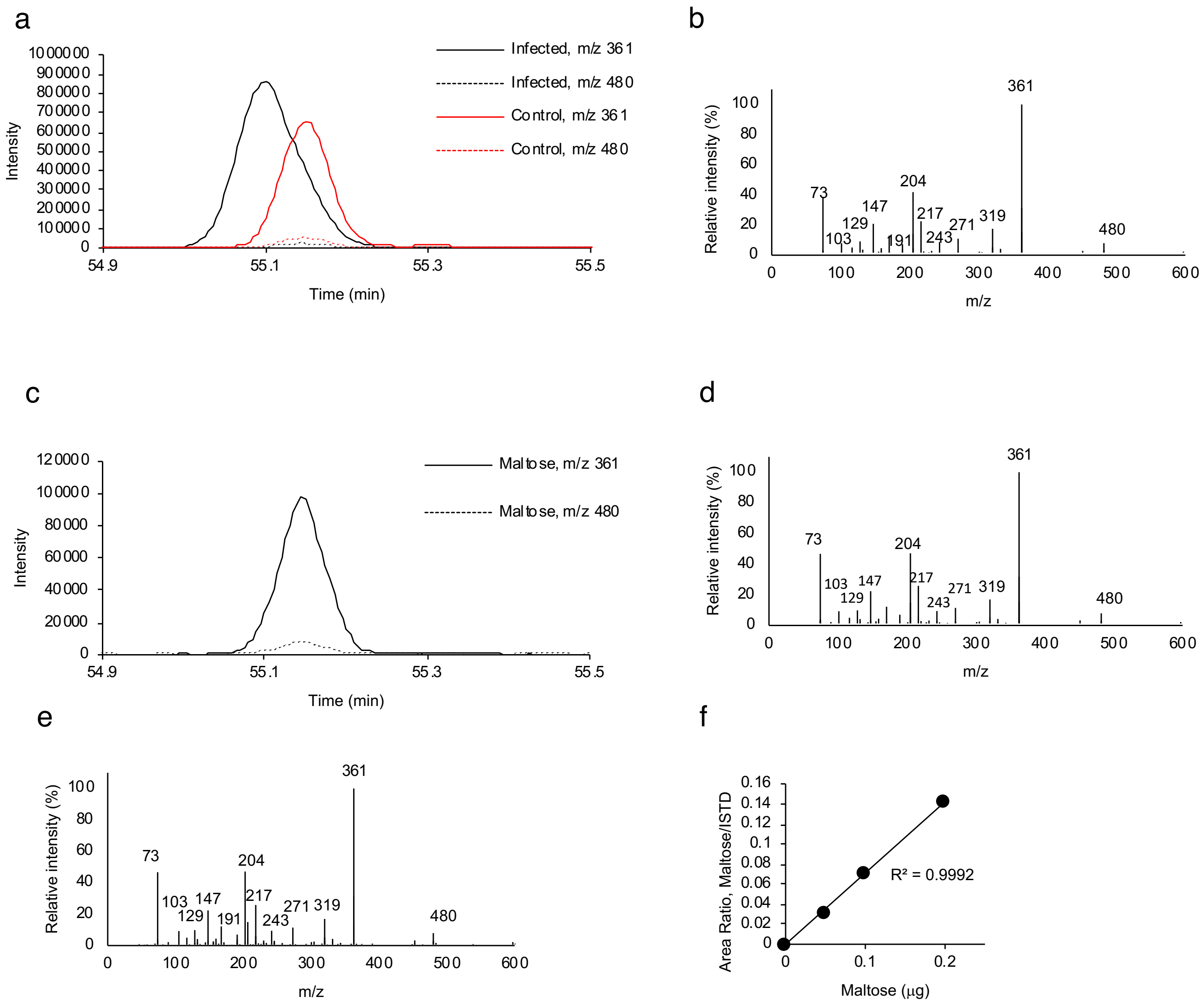

Supplementary Figure 6. Chromatographic and mass spectrometric distinction of maltose from trehalose. Intestinal contents of infected and control mice (**a**), and 500 ng maltose (**c**) were analysed using the GC/MS full-scan mode (m/z 45 - 600) with methoximation. Representative mass chromatograms (m/z 361and 480) of an infected sample indicated that at least two compounds were present. It was likely that the peak at 55.100 min was derived from trehalose (**Supplementary Fig. 5**). However, the peak at 55.150 in the control sample was dominant in the control sample and not in the infected sample. (**b**) Mass spectrum of the peak corresponding to the control sample in **a** had high similarity with that of methoximated maltose (**d**). Mass chromatogram (m/z 361) showed that the retention times were very similar among **a** and **c**, suggesting that the peak at 55.150 min in (**a**) was derived from maltose. (**d**) Mass spectrum of the peak in (**c**) had a distinctive fragment ion of m/z 480, which was not present in the mass spectrum of trehalose. (**e**) Standard mass spectrum of methoximated maltose collected in the spectrum library of the GCMS solution based on NIST11. (**f**) Maltose was analysed using the GC/MS full-scan mode with methoximation. The fragment ion m/z 480 specific for maltose was used for the quantification. 2-IPM (0.5 μg) was used for the internal standard. All experiments were repeated two times with similar results.

Supplementary Figure 7

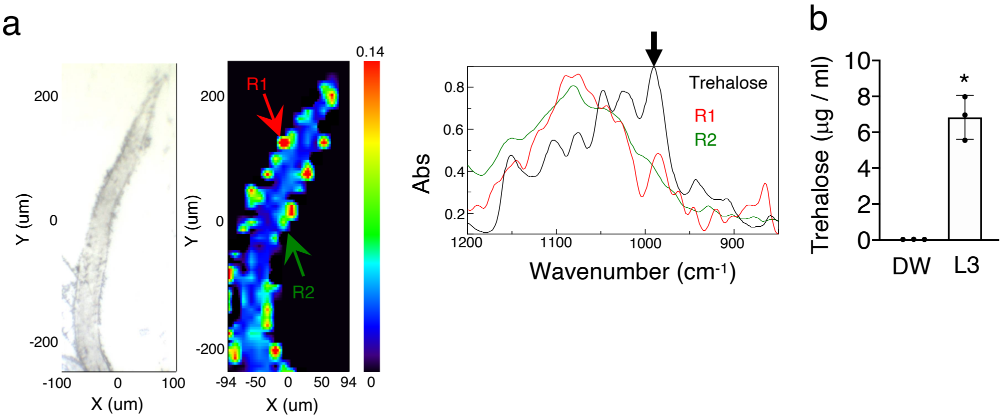

Supplementary Figure 7. Secretion of trehalose from infective L3 larvae of Hp.

(**a**) As indicated in the spectrum of pure trehalose crystals (black line in the right panel), this sugar had a unique vibration band at 992 cm^{-1} (arrow), which reflected an α,α-1,1 bond between two glucose molecules. FITR microscopic observations using this peak were performed to visualize the location of trehalose in L3 larvae. The resulting FTIR image is shown in the centre panel together with the corresponding bright field image (left panel). The image size is approximately 200 µm wide and 500 µm long. The peak intensity at 992 cm^{-1} was expressed along with the colour scale from the darkest red to the lightest violet. The concentrated trehalose regions were located along the surface of the worm body. The FTIR spectra of R1 and R2 regions in the FTIR image are shown in the right panel. The spectrum of the R1 region exhibited a distinct peak at 992 cm^{-1}, which confirmed the existence of trehalose. (**b**) Trehalose concentrations in the preservative solution in the absence (DW) or presence of 200 L3 larvae/ml were measured using a trehalose assay kit. Values represent the mean ± SD from triplicate cultures. Asterisks denote statistical significance at $p < 0.01$ calculated by the two-tailed Mann-Whitney test. FTIR observation was performed once and treahalose concentrations were measured three times with similar results.

Supplementary Figure 8

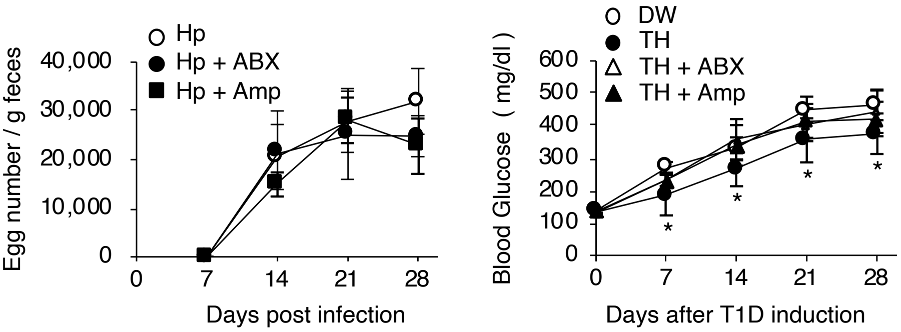

Supplementary Figure 8. No adverse effects of antibiotics on Hp infection. Kinetics of Hp infection in mice treated with ABX or Amp were monitored by counting eggs in faeces. Values represent the mean ± SD of 10 mice. Asterisks denote statistical significance at $p < 0.05$ calculated by the two-way ANOVA. All experiments were repeated at least three times with similar results.

Supplementary Figure 9

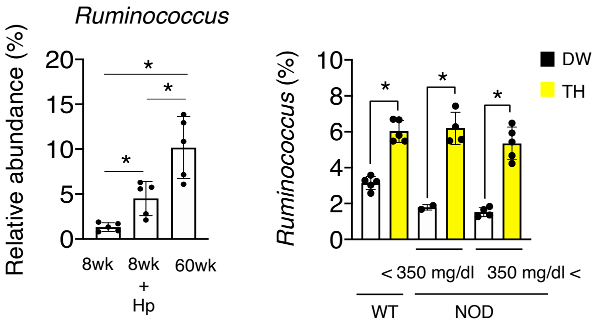

Supplementary Figure 9. Abundance of *Ruminococcus* in aged C57BL/6 and NOD mice. Abundance of *Ruminococcus* in aged mice (N=5) and NOD mice used in **Fig. 2l** was evaluated as described in **Fig. 3f**. Values represent the mean ± SD. Asterisks denote statistical significance at $p < 0.05$ calculated by the Tukey post-hoc analysis. Experiments using aged mice were repeated at least three times with similar results and those using NOD mice were performed once.

Supplementary Figure 10

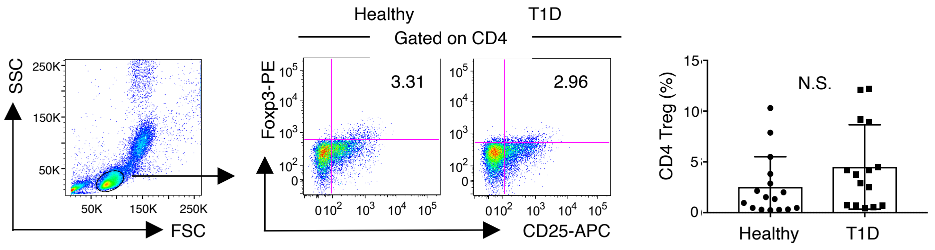

Supplementary Figure 10. CD4Tregs in T1D patients. Cells from T1D patients (N=15) and healthy volunteers (N=15) used in **Fig. 4** were also analysed for CD4Tregs. The gated $CD4^+$ cells were plotted onto CD25 and Foxp3 (left panels). The numbers indicate the percentages of $CD25^+Foxp3^+$ CD4Tregs among the gated $CD4^+$ cells. The frequency of CD4Tregs is presented as described in **Figure 4b** (right panels). Values represent the mean ± SD. NS indicates non-significant using the two-sided unpaired Student's *t*-test. All experiments using human samples were performed once.

Supplementary Figure 11

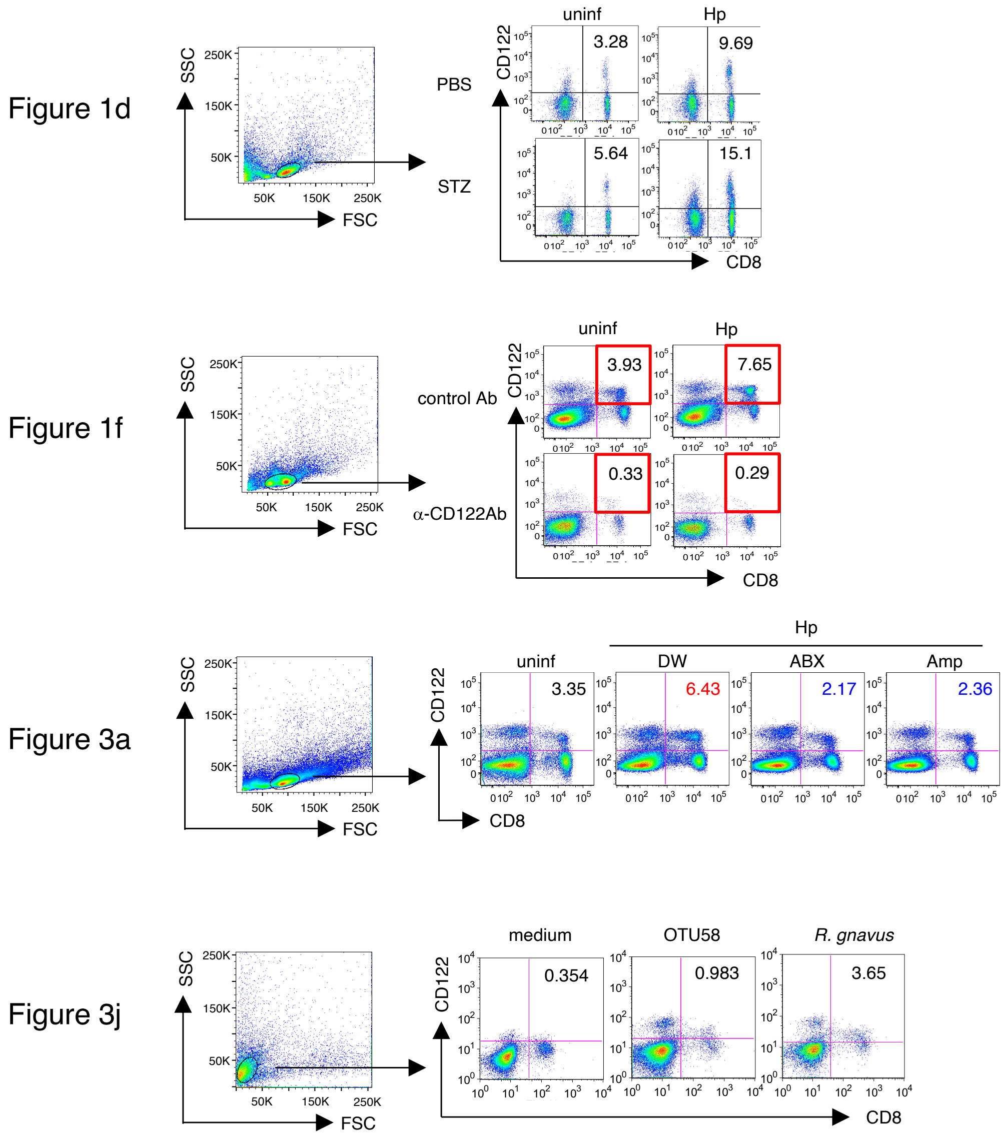

Supplementary Figure 11 Gating strategies for Fig. 1d, f, and Fig. 3a, j are shown.

Supplementary Table 1: Metabolites identified in the intestinal contents

	Metabolite name	Average normalized area (5 controls)	Average normalized area (4 infected)	Area ratio (Infected/Control)	*p* value	Bonferroni ($p < 0.001$)	$\log_2$(FC)	$-\log_{10}$(p)	**p*, BH	transition (m/z)	%CV (QC)	RT (min)	ΔRT(min)
1	Trehalose-8TMS	4,140	73,778	17.82	3.2E-04	significant	4.16	3.49	* 0.015	361.10>73.00	4.39	55.03	0.099
2	Asparagine-3TMS	928	2,605	2.81	2.6E-03		1.49	2.58	* 0.062	188.10>73.00	6.40	29.89	0.059
3	Glycerol 3-phosphate-4TMS	2,024	11,616	5.74	7.1E-02		2.52	1.15	1.13	357.10>73.00	5.69	32.57	0.047
4	5-Aminovaleric acid-3TMS	751	1,305	1.74	9.9E-02		0.80	1.00	1.19	174.20>73.00	7.20	28.65	0.091
5	Ascorbic acid-4TMS	70	1,544	22.15	0.1129		4.47	0.95	1.08	319.10>73.00	2.95	37.59	0.122
6	2-Aminoethanol-3TMS	712	1,515	2.13	0.1792		1.09	0.75	1.43	174.10>73.00	17.98	16.99	0.075
7	Mannitol-6TMS	45,419	4,728	0.10	0.1792		-3.26	0.75	1.23	319.20>73.00	2.13	37.39	0.093
8	4-Aminobutyric acid-3TMS	543	1,295	2.39	0.2485		1.25	0.60	1.49	174.10>73.00	8.28	25.71	0.081
9	Glycine-2TMS	1,338	370	0.28	0.2776		-1.86	0.56	1.48	176.10>147.10	28.85	11.59	0.098
10	Phosphoric acid-3TMS	33,525	69,766	2.08	0.2801		1.06	0.55	1.34	299.10>73.00	1.35	17.16	0.118
11	Isoleucine-2TMS	36,259	16,779	0.46	0.2890		-1.11	0.54	1.26	158.20>73.00	0.94	17.89	0.124
12	Creatinine-3TMS	342	604	1.77	0.2912		0.82	0.54	1.16	329.20>115.10	1.53	26.71	0.094
13	Taurine-3TMS	2,182	277	0.13	0.3015		-2.98	0.52	1.11	174.10>73.00	22.74	30.05	0.104
14	Leucine-2TMS	102,601	52,331	0.51	0.3084		-0.97	0.51	1.06	158.10>73.00	2.16	17.09	0.120

15	Valine-2TMS	49,342	25,724	0.52	0.3284		-0.94	0.48	1.05	144.10>73.00	1.28	15.14	0.107
16	Histidine-3TMS	13,250	6,384	0.48	0.3665		-1.05	0.44	1.10	154.10>73.00	10.61	36.77	0.092
17	Phenylalanine-2TMS	45,978	27,340	0.59	0.4088		-0.75	0.39	1.15	218.10>73.00	1.67	28.74	0.064
18	Glutamine-3TMS	6,552	9,344	1.43	0.4128		0.51	0.38	1.10	245.10>156.10	9.06	32.73	0.086
19	Ornithine-3TMS	8,837	4,889	0.55	0.4400		-0.85	0.36	1.11	142.10>73.00	15.82	28.20	0.090
20	Tyrosine-3TMS	117,126	75,282	0.64	0.4807		-0.64	0.32	1.15	218.10>73.00	1.30	37.20	0.089
21	Sorbitol-6TMS	4,195	2,446	0.58	0.4866		-0.78	0.31	1.11	203.10>73.00	3.10	37.67	0.068
22	Tryptophan-3TMS	46,086	30,628	0.66	0.4935		-0.59	0.31	1.08	202.10>73.00	1.97	44.03	0.091
23	Stearic acid-TMS	19,004	23,478	1.24	0.4981		0.30	0.30	1.04	145.10>129.10	4.22	43.89	0.073
24	Aspartic acid-3TMS	39,783	25,286	0.64	0.5142		-0.65	0.29	1.03	232.10>73.00	1.12	25.35	0.072
25	Serine-3TMS	20,517	14,261	0.70	0.5215		-0.52	0.28	1.00	204.10>73.00	1.46	20.16	0.064
26	Lactic acid-2TMS	49,206	85,170	1.73	0.5437		0.79	0.26	1.00	191.10>147.10	3.63	9.59	0.101
27	Proline-2TMS	12,459	9,026	0.72	0.5446		-0.46	0.26	0.97	142.10>73.00	4.11	18.09	0.092
28	Glutamic acid-3TMS	23,870	16,698	0.70	0.5560		-0.52	0.25	0.95	246.10>73.00	2.20	28.32	0.082
29	Palmitic acid-TMS	9,890	11,789	1.19	0.5691		0.25	0.24	0.94	313.30>75.10	5.48	39.37	0.063
30	Arginine-3TMS	2,153	1,566	0.73	0.5964		-0.46	0.22	0.95	142.10>73.00	6.88	34.08	0.090
31	Glyceric acid-3TMS	1,235	878	0.71	0.6011		-0.49	0.22	0.93	189.10>73.00	1.76	19.14	0.145
32	Threonine-3TMS	7,249	5,503	0.76	0.6102		-0.40	0.21	0.92	218.10>73.00	1.11	21.11	0.082
33	Fumaric acid-2TMS	537	701	1.30	0.6284		0.38	0.20	0.91	241.10>147.10	2.18	19.41	0.112
34	Sucrose-8TMS	18,149	28,649	1.58	0.6374		0.66	0.20	0.90	361.10>73.00	7.58	53.04	0.154
35	5-Oxoproline-2TMS	101,934	75,939	0.74	0.7049		-0.42	0.15	0.97	156.10>73.00	3.98	25.51	0.070
36	Glycine-3TMS	3,919	4,576	1.17	0.7272		0.22	0.14	0.97	174.10>73.00	5.27	18.42	0.100

37	Hypoxanthine-2TMS	1,436	1,151	0.80	0.7405		-0.32	0.13	0.96	265.10>73.00	1.68	33.70	0.106
38	Niacinamide-TMS	433	541	1.25	0.7534		0.32	0.12	0.95	179.10>75.00	1.42	24.00	0.086
39	Lysine-4TMS	5,361	6,116	1.14	0.7645		0.19	0.12	0.94	317.20>156.20	11.04	36.72	0.095
40	Alanine-2TMS	76,523	66,099	0.86	0.7882		-0.21	0.10	0.95	116.10>73.00	2.35	11.07	0.092
41	Ornithine-4TMS	4,530	4,013	0.89	0.8539		-0.17	0.07	1.00	157.20>73.00	9.06	34.10	0.070
42	Adenosine-4TMS	2,397	2,088	0.87	0.8734		-0.20	0.06	1.00	230.10>73.00	4.81	52.46	0.087
43	Cysteine-3TMS	5,080	5,479	1.08	0.8789		0.11	0.06	0.98	220.10>73.00	10.26	26.52	0.096
44	Uracil-2TMS	684	630	0.92	0.8871		-0.12	0.05	0.97	245.00>147.10	1.56	19.44	0.051
45	Succinic acid-2TMS	1,106	1,020	0.92	0.8887		-0.12	0.05	0.95	247.10>147.10	0.81	18.36	0.052
46	Glycerol-3TMS	3,521	3,705	1.05	0.9286		0.07	0.03	0.97	133.10>73.00	2.85	17.14	0.108
47	Xanthine-3TMS	914	884	0.97	0.9626		-0.05	0.02	0.98	353.10>147.10	3.21	39.21	0.081
48	Inosine-4TMS	842	846	1.01	0.9937		0.01	0.00	0.99	259.10>73.00	4.44	51.25	0.085

Supplementary table 1

Metabolites in the intestinal contents (5 controls and 4 infected) were analyzed using GC/MS MRM mode. Forty-eight metabolites were identified manually using GC/MS solution software version 4.41 (Shimadzu). Each area of metabolite was normalized by the area of 2-IPM and the weight of intestinal content. Intra-assay variability was examined by peak areas of 2-IPM in all the measurements. Its %CV value was 8.9%, which showed the measurement was stable. The p values were calculated with the two-tailed unpaired Student's t-test. Metabolites were sorted according to the p values. Among the 48 metabolites, only the trehalose level was significantly high in the infected group with the significance level of 0.05 when it was adjusted with Bonferroni's method ($p < 0.001$). When false discovery rate (FDR) was considered using Benjamini and Hochberg (BH) method, the aspargine level, in addition to trehalose, was also

significantly high in the infected group with FDR 0.1, which was shown as asterisks.

Intra-assay variability was also examined using quality control (QC) samples. The QC sample that was prepared by mixing all the target samples (n = 9) was processed in the same way as the target samples. Four independent measurements were done for the QC samples. Concerning the QC measurements, the %CV values of peak areas that were normalized by the areas of 2-IPM, indicated that the measurement was stable enough for most of the metabolites.

All the retention times for the identified metabolites were close to the set values.

Supplementary Table 2: Metabolites identified in the HES/DEMEM

	Metabolite name	Average normalized area (DMEM)	Average normalized area (HES)	Area ratio (HES/Cont)	*p* value	Bonferroni ($p < 0.0015$)	$\log_2$(FC)	$-\log_{10}$(p)	**p*, BH	transition (m/z)	%CV (QC)	RT (min)	ΔRT(min)
1	Trehalose-8TMS	365	65,149	178.52	1.6E-06	significant	7.48	5.80	* 5.2E-05	361.10>73.00	3.19	55.04	0.095
2	Succinic acid-2TMS	1,006	8,474	8.43	4.8E-06	significant	3.08	5.32	* 7.9E-05	247.10>147.10	3.35	18.36	0.051
3	Hydroquinone-2TMS	12,410	14,557	1.17	5.1E-04	significant	0.23	3.30	* 5.6E-03	254.10>239.10	1.06	21.49	0.091
4	Lactic acid-2TMS	84,537	283,657	3.36	1.7E-03		1.75	2.78	* 0.014	191.10>147.10	13.04	9.59	0.100
5	3-Hydroxypropionic acid-2TMS	2,135	2,654	1.24	0.0023		0.31	2.64	* 0.015	177.10>147.10	3.02	12.43	0.104
6	Alanine-2TMS	8,116	45,752	5.64	0.0056		2.49	2.25	* 0.031	116.10>73.00	21.42	11.07	0.092
7	Proline-2TMS	834	12,888	15.45	0.0076		3.95	2.12	* 0.036	142.10>73.00	26.58	18.09	0.092
8	Glyceric acid-3TMS	7,910	9,378	1.19	0.0083		0.25	2.08	* 0.034	189.10>73.00	3.33	19.14	0.145
9	Phenylacetic acid-TMS	4,587	5,132	1.12	0.0293		0.16	1.53	0.11	164.10>73.00	2.22	17.99	0.060
10	Nonanoic acid-TMS	27,605	30,075	1.09	0.1594		0.12	0.80	0.53	215.10>75.00	6.04	19.91	0.015
11	Niacinamide-TMS	27,346	25,887	0.95	0.2247		-0.08	0.65	0.67	179.10>75.00	4.08	24.00	0.088
12	Glycolic acid-2TMS	7,578	8,262	1.09	0.2547		0.12	0.59	0.70	177.10>147.10	3.70	10.05	0.065
13	Phosphoric acid-3TMS	68,012	70,028	1.03	0.3464		0.04	0.46	0.88	299.10>73.00	2.25	17.16	0.121
14	Histidine-3TMS	70,994	64,747	0.91	0.3539		-0.13	0.45	0.83	154.10>73.00	8.97	36.77	0.095
15	Methionine-2TMS	4,502	4,868	1.08	0.3878		0.11	0.41	0.85	250.10>147.10	9.71	25.37	0.083
16	Tyrosine-3TMS	1,313,551	1,262,184	0.96	0.4021		-0.06	0.40	0.83	218.10>73.00	5.06	37.20	0.093

17	Octadecanol-TMS	6,545	7,583	1.16	0.4315		0.21	0.37	0.84	327.30>75.00	21.06	41.92	0.073
18	Tryptophan-3TMS	278,796	251,759	0.90	0.4520		-0.15	0.34	0.83	202.10>73.00	12.57	44.02	0.096
19	Valine-2TMS	1,647,087	1,456,075	0.88	0.4643		-0.18	0.33	0.81	144.10>73.00	17.82	15.14	0.105
20	Phenylalanine-2TMS	478,821	455,954	0.95	0.4838		-0.07	0.32	0.80	218.10>73.00	7.60	28.74	0.066
21	5-Oxoproline-2TMS	14,809,609	14,113,417	0.95	0.4873		-0.07	0.31	0.77	156.10>73.00	6.65	25.51	0.070
22	Isoleucine-2TMS	1,547,361	1,400,300	0.90	0.5228		-0.14	0.28	0.78	158.20>73.00	16.82	17.89	0.123
23	Leucine-2TMS	1,870,530	1,653,445	0.88	0.5273		-0.18	0.28	0.76	158.10>73.00	20.71	17.09	0.119
24	Arginine-3TMS	10,651	8,865	0.83	0.6054		-0.26	0.22	0.83	157.20>73.00	11.87	34.10	0.072
25	Caproic acid-TMS	5,379	5,043	0.94	0.6191		-0.09	0.21	0.82	173.10>75.00	10.47	9.93	0.087
26	Threonine-3TMS	290,525	272,279	0.94	0.6437		-0.09	0.19	0.82	218.10>73.00	14.90	21.11	0.083
27	Glutamine-3TMS	52,751	50,207	0.95	0.6523		-0.07	0.19	0.80	245.10>156.10	8.77	32.73	0.088
28	Lysine-4TMS	39,965	35,178	0.88	0.6575		-0.18	0.18	0.77	317.20>156.20	25.09	36.72	0.099
29	Serine-3TMS	407,157	382,657	0.94	0.7002		-0.09	0.15	0.80	204.10>73.00	17.46	20.16	0.066
30	Glycine-3TMS	47,680	43,473	0.91	0.7173		-0.13	0.14	0.79	174.10>73.00	20.88	18.42	0.098
31	Benzoic acid-TMS	13,663	14,217	1.04	0.7817		0.06	0.11	0.83	179.10>105.10	15.77	16.23	0.075
32	Ornithine-4TMS	13,194	13,918	1.05	0.8112		0.08	0.09	0.84	142.10>73.00	18.77	34.09	0.084
33	Pantothenic acid-3TMS	9,476	9,699	1.02	0.8531		0.03	0.07	0.85	291.20>201.10	8.19	38.58	0.094

Supplementary table 2

Metabolites in HES and DMEM (3 HES and 3 DMEM) were analyzed using GC/MS MRM mode. Thirty-three metabolites were identified manually using GCMS solution software version 4.41 (Shimadzu). Each area of metabolite was normalized by the area of 2-IPM. Intra-assay variability was examined by

peak areas of 2-IPM in all the measurements. Its %CV value was 19%. The *p* values were calculated with the two-tailed unpaired Student's t-test. Metabolites were sorted according to the *p* values. Among the 33 metabolites, the levels of three metabolites, trehalose, succinic acid and Hydroquinone, were significantly high in HES group with the significance level of 0.05 when it was adjusted with Bonferroni's method ($p < 0.0015$). When FDR was considered using BH method, 8 metabolites were significantly high in the infected group with FDR 0.1, which was shown as asterisks.

Intra-assay variability was also examined using QC samples. The QC sample that was prepared by mixing HES and DMEM samples was processed in the same way as the target samples. Four independent measurements were done for the QC samples. Concerning the QC measurements, the %CV values of peak areas that were normalized by the areas of 2-IPM, indicated that the measurement was stable enough for most of the metabolites. All the retention times for the identified metabolites were close to the set values.

Supplementary Table 3. Summary of clinical characteristics of the T1D participants and healthy volunteers.

	Number	Sex	Age	Duration from onset	Blood sugar (mg/dl)	HbA1c (%)	RBC (10^6 /ul)	WBC (10^3 /ul)	Platelet (10^4 / ul)	Anti-GAD[1] antibody (U/ml)[2,3]	Anti-IA-2 antibody[2,4]	Anti-Insulin Antibody[2,5]	CD8 Treg (%)	CD4 Treg (%)
Healthy	1	F	2	-	-	-	-	-	-	-			1.93	2.15
	2	F	2	-	-	-	-	-	-	-			1.55	0.464
	3	M	7	-	-	-	-	-	-	-			3.76	0.475
	4	F	10	-	-	-	-	-	-	-			6.88	0.32
	5	F	10	-	-	-	-	-	-	-			4.28	0.297
	6	F	10	-	-	-	-	-	-	-			3.48	0.276
	7	F	10	-	-	-	-	-	-	-			2.55	0.249
	8	F	12	-	-	-	-	-	-	-			11.7	2.87
	9	F	13	-	-	-	-	-	-	-			5.32	1.53
	10	M	13	-	-	-	-	-	-	-			4.99	1.34
	11	F	13	-	-	-	-	-	-	-			4.02	0.962
	12	M	14	-	-	-	-	-	-	-			2.84	10.3
	13	M	14	-	-	-	-	-	-	-			5.69	7.88
	14	M	14	-	-	-	-	-	-	-			6.17	5.49
	15	F	16	-	-	-	-	-	-	-			7.59	3.821
	16	F	16	-	-	-	-	-	-	-			13.4	2.001
T1D	1	F	2	0mo	230	7.1	448	5.6	29.1	8.2			3.6	9.18
	2	F	3	2yr 11mo	157	8.5	486	11.3	44.9	26			2.28	0.45
	3	F	6	1yr 4mo	112	8.6	468	8.6	34.1	> 2000			2.89	4.47
	4	F	7	1mo	137	10.5	461	3.3	21.4	52	< 0.4	< 125	3.19	12.1
	5	F	9	1yr 10mo	111	8.7	489	4.1	28.2	+	+		3.11	8.96
	6	F	10	1yr 9mo	306	7.9	494	6.1	27.2	0.5	< 0.4	< 125	2.44	0.73
	7	F	10	7yr 7mo	261	8.1	422	5.7	17.8	0.7	8.6		1.21	0.53
	8	F	11	1yr 5mo	162	7.8	520	6.2	27.5	10.8			2.62	4.22
	9	M	14	7yr 11mo	175	7	496	5.8	21.8	36	9.8		0.776	2.51
	10	F	14	8yr 11mo	41	7.2	435	6.2	26.9	1.7	18	5.2	2.85	2.51
	11	M	15	14yr 7mo	88	8.9	506	5.3	20.2	18.5			1.37	1.99
	12	F	15	6yr 2mo	64	7.5	426	5.8	20.7	101	< 0.4		2.59	4.18
	13	M	15	1yr 7mo	104	5.9	511	6.4	22.8	10.3	11		1.97	2.9
	14	M	15	4yr 7mo	89	7	504	6.2	24.6	64			2.35	3.73
	15	M	15	4yr 7mo	137	6.3	550	3.7	18.1	3.1			2.66	12.2

[1]glutamic acid decarboxylase, [2]values on the diagnosis, [3]normal range < 1.5 U/ml, [4]normal range < 0.4 U/ml, [5]normal range < 125 nU/ml, [6]not determined
